# Systemic treatment in EGFR-ALK NSCLC patients: second line therapy and beyond

**DOI:** 10.7497/j.issn.2095-3941.2014.03.003

**Published:** 2014-09

**Authors:** Niki Karachaliou, Rafael Rosell

**Affiliations:** ^1^Translational Research Unit, Dr Rosell Oncology Institute, Quirón Dexeus University Hospital, 08028 Barcelona, Spain; ^2^Cancer Biology and Precision Medicine Program, Catalan Institute of Oncology, Hospital Germans Trias i Pujol, Ctra Canyet s/n, 08916 Badalona, Barcelona, Spain; ^3^Fundación Molecular Oncology Research (MORe), Sabino Arana 5-19, 08028 Barcelona, Spain

**Keywords:** Lung cancer, epidermal growth factor (*EGFR*), anaplastic lymphoma kinase fusions (*ALK* fusions), tyrosine kinase inhibitors (TKIs), TKI resistance

## Abstract

Lung cancer is the most frequently diagnosed cancer and a leading cause of cancer mortality worldwide, with adenocarcinoma being the most common histological subtype. Deeper understanding of the pathobiology of non-small cell lung cancer (NSCLC) has led to the development of small molecules that target genetic mutations known to play critical roles in progression to metastatic disease and to influence response to targeted therapies. The principle goal of precision medicine is to define those patient populations most likely to respond to targeted therapies. However, the cancer genome landscape is composed of relatively few “mountains” [representing the most commonly mutated genes like KRAS, epidermal growth factor (EGFR), and anaplastic lymphoma kinase (ALK)] and a vast number of “hills” (representing low frequency but potentially actionable mutations). Low-frequency lesions that affect a druggable gene product allow a relatively small population of cancer patients for targeted therapy to be selected.

## Introduction

In recent years, diagnosis and treatment of patients with advanced lung cancer have undergone transformational changes. The current paradigm for prescribing novel targeted therapies is based on selecting patients according to the presence of specific oncogenic abnormalities in the tumor. The efficacy of therapy targeted at a specific oncogene is convincing evidence of “oncogene addiction”, or the concept that some cancers rely on or are “addicted to” a specific gene for their survival and proliferation^1^.

The first such abnormalities discovered in lung cancer were epidermal growth factor (*EGFR*) kinase domain mutations; tumors with these mutations were found to be sensitive to EGFR tyrosine kinase inhibitors (TKIs)^2^. The echinoderm microtubule-associated protein-like 4- anaplastic lymphoma kinase (EML4-ALK) fusion has emerged as the second most important driver oncogene in lung cancer and the first targetable fusion oncokinase to be identified in 4%-6% of lung adenocarcinomas^3^^,^^4^. Crizotinib, an oral small-molecule inhibitor of ALK and c-MET receptor kinases, is now approved for treatment of *ALK* positive advanced non-small cell lung cancer (NSCLC), based on the results of two pivotal studies^5^^-^^7^.

Among *EGFR* or *ALK* mutated NSCLCs, the percentage of complete response is negligible and therefore novel, effective, safe treatments need to be tested and developed. To this end, repurposing an existing drug for treatment of NSCLC is also a worthy goal.

### EGFR NSCLC patients: second line therapy and beyond

Our group was the first to evaluate the feasibility of large-scale screening for *EGFR* mutations in patients with advanced NSCLC and analyze the association between the mutations and the outcome of erlotinib treatment^8^. Since the introduction of erlotinib and gefitinib, patients with metastatic *EGFR* positive lung cancer can be offered a therapeutic alternative that has proven its superiority over standard platinum-based chemotherapy^2^^,^^9^. In the EURTAC study, in which erlotinib was compared with platinum-doublet chemotherapy as first-line treatment for patients with *EGFR*-mutant NSCLC, erlotinib demonstrated a significant improvement in the overall response rate (ORR) and median progression free survival (PFS)^2^. On the basis of this study, in May 2013 the U.S Food and Drug Administration (FDA) approved erlotinib for use in patients with lung cancers harboring *EGFR* exon 19 deletions and EGFR L858R substitutions^2^.

Whereas reversible EGFR TKIs compete with ATP in the kinase domain of *EGFR*, 2^nd^ generation EGFR TKIs, also compete for ATP binding but then covalently bind at the edge of the ATP binding cleft on Cys773 of *EGFR* via the Michael mechanism (addition of nucleophile to an α, β unsaturated carbonyl)^10^. Currently there are two lead 2^nd^ generation EGFR TKI candidates, afatinib and dacomitinib, that are active against *EGFR* mutations with acquired resistance to erlotinib or gefitinib^11^. Afatinib exhibits superior anticancer activity in lung cancer patients harboring gefitinib/erlotinib-resistant mutant *EGFR* (including T790M, exon 20 insertion, and T790M/L858R double mutation)^12^. Based on the results of the LUX-Lung 3, the FDA has approved afatinib as a new first-line treatment for patients with metastatic *EGFR*-mutated NSCLC^13^. In the pooled analysis from LUX-Lung 3 and 6, presented this year at ASCO, afatinib prolonged survival of lung cancer patients whose tumors have common EGFR mutations by a median of three months compared with standard chemotherapy and significantly reduced the risk of death by 19%. The most pronounced reduction in risk of death was 41% in patients whose tumors had the most common exon 19 deletion EGFR mutation; for patients with the L8585R mutation there was no impact on overall survival^14^.

Despite the recent paradigm shift in the treatment of NSCLC patients, with a move toward biomarker-directed therapy, preclinical data has seldom been incorporated into clinical practice, since the existence of multiple resistance mechanisms complicates the selection of optimal biomarkers. Among patients progressing to first generation EGFR TKIs, 50% have tumors with a secondary T790M mutation^15^. The emergence of the T790M *EGFR* gatekeeper mutation and up-regulation of downstream signaling by *MET* amplification have been described as the two main mechanisms responsible for acquired resistance^16^. However a phase III trial enrolling only patients with *MET*-positive tumors was stopped in early March 2014 due to futility; there was no evidence to suggest a positive effect of addition of onartuzumab to erlotinib^17^.

Other mechanisms include *EGFR* amplifications, *PI3KCA* mutations or a transition from epithelial to mesenchymal differentiation. For a small percentage of resistant tumors, histological transformation occurs to small cell lung cancer (SCLC)^16^. We recently reported possible causes of resistance to *EGFR* TKIs in *EGFR*-mutant NSCLC patients: the high co-existence of the pretreatment somatic T790M mutation, with a clear impact on PFS, and the role of *BIM* mRNA expression as an independent prognostic marker^18^. Pretreatment T790M can be detected in more than 60% of cases^18^. In fact, using a PCR-PNA assay pretreatment, T790M mutations were detected in 65.26% of patients^18^. These results reinforce the need for 2^nd^ and 3^rd^ generation EGFR TKIs, while taking into account existing data that suggest use of erlotinib or gefitinib beyond progression, with added chemotherapy, radiotherapy or best supportive care may improve survival^19^. Although afatinib and dacomitinib have been introduced to overcome acquired resistance, they showed limited efficacy in NSCLC with T790M and were more than 100-fold less potent in NSCLC cells with *EGFR* T790M mutation than in NSCLC cells with *EGFR* activating mutation^20^.

CO-1686 is a novel covalent inhibitor that irreversibly and selectively targets both the initial activating *EGFR* mutations and the T790M secondary acquired resistance mutation^21^. To investigate its use as a single agent, CO-1686 is being evaluated in a phase I/II trial in *EGFR*-mutant NSCLC patients previously treated with first-line gefitinib or erlotinib (NCT01526928)^22^. In the phase I study, and based on early findings from the ongoing phase II trial, the agent yielded a 58% ORR across all dose levels in trial participants with biopsy-confirmed *EGFR* T790M mutations. Additionally, the compound did not cause the skin rash and diarrhea commonly associated with earlier generations of *EGFR* inhibitors^23^. AZD9291 showed high activity in preclinical studies and was well tolerated in animal models. Xenograft studies identified a breakdown metabolite of AZD9291 called AZ5104 that is about five times as potent as AZD9291 itself^24^. In the phase I study of AZD9291 in *EGFR* mutant patients resistant to standard EGFR TKIs, 50% of patients experienced tumor shrinkage and the drug worked particularly well in patients with the T790M mutation^25^.

A consistent proportion of *EGFR* mutant patients, approximately 30%, never respond to EGFR TKIs due to primary resistance. *BIM* mRNA expression could enrich the molecular diagnosis in patients with *EGFR* mutations, by identifying patients unlikely to respond and those with stable disease for whom treatment may not be effective due to early adaptive resistance^18^^,^^26^. It is worth mentioning that, besides *BIM* expression, a common BIM deletion polymorphism impairs generation of the proapoptotic isoform required for EGFR TKIs^27^. This polymorphism confers an inherent drug-resistant phenotype that can be circumvented with the addition of the histone deacetylase (HDAC) inhibitor vorinostat to EGFR TKIs^28^.

The two primary signaling pathways activated by *EGFR* include the mitogen-activated protein kinase (MAPK), and the phosphoinositide-3-kinase (PI3K), axes. Src tyrosine kinases and activation of the signal transducer and activator of transcription 3 (STAT3) pathways and downstream signaling have also been well documented^29^. EGFR phosphorylation leads to recruitment of multiple effector proteins through recognition and binding of Src-homology 2 domain-containing phosphatase 2 (SHP2) to phosphotyrosine motifs on the receptor^29^. SHP2 (encoded by *PTPN11*) is a ubiquitously expressed SH2 domain-containing protein tyrosine phosphatase (PTP). Despite its direct function in protein dephosphorylation, SHP2 plays an overall positive role in transducing signals initiated from growth factors/cytokines and extracellular matrix proteins, and initiating various downstream signaling cascades, including the PI3K and MAPK^29^^,^^30^. By contrast, SHP2 functions as a negative regulator of the JAK/STAT pathway^31^.

In 2004, Sordella and colleagues were able to demonstrate the differential EGF-induced tyrosine phosphorylation pattern seen with wild-type (WT) and mutant EGFR receptors^32^. For instance, Y845 is highly phosphorylated in the L858R missense mutant, but not in the WT or deletion mutant, and hence appears to be unique in distinguishing between the two types of *EGFR* mutations^32^. Y845 (pY845) phosphorylation stabilizes the activation loop, maintains the enzyme in an active state, and regulates STAT3/5 activity^32^. Surprisingly, the *EGFR* L858R mutation leads to decreased ability to activate ERK compared to WT *EGFR* which correlates with decreased EGFR internalization, reduced phosphorylation of SHP2, hyperactivity of STAT3 and reduced sensitivity to gefitinib^33^. Lazzara and colleagues found that SHP2 Y542 phosphorylation was not induced in response to EGF in the H3255 cells, which harbor the missense L858R exon 21 mutation, suggesting that SHP2 activity may be less efficiently promoted by *EGFR* L858R and the STAT3 pathway may be more active^34^. The main problem is that STAT3 activation is not abrogated by single EGFR TKI treatment with gefitinib, erlotinib, afatinib or dacomitinib^20^^,^^34^^-^^36^. Indeed, the second generation irreversible *EGFR* TKIs, afatinib or dacomitinib, not only do not abrogate, but may even induce STAT3 phosphorylation in gefitinib or erlotinib resistant cell lines such as H1975 or PC9-R20.

**Figure 1:**
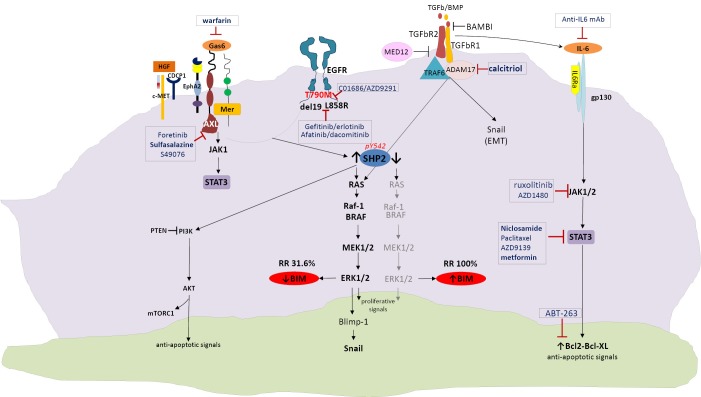
Crosstalk signaling pathways as potential mechanisms of resistance to reversible or irreversible EGFR TKIs. EGFR, epidermal growth factor; TKIs, tyrosine kinase inhibitors.

Afatinib activates interleukin-6 receptor (IL-6R)/JAK1/STAT3 signaling via autocrine IL-6 secretion in both cells. Blockade of IL-6R/JAK1 significantly increased sensitivity to afatinib through inhibition of afatinib-induced STAT3 activation. The role of the paracrine IL-6R/JAK1/STAT3 loop between stroma and cancer cells in the development of drug resistance is crucial^20^. Yao *et al*. uncovered the existence of a subpopulation of cells intrinsically resistant to erlotinib which display features suggestive of epithelial-to-mesenchymal transition (EMT) in NSCLC-derived cell lines and early-stage tumors before erlotinib treatment^37^. Activation of TGF-beta-mediated signaling was sufficient to induce these phenotypes. An increased TGF-beta-dependent IL-6 secretion released previously addicted lung tumor cells from their EGFR dependency. Therefore, both tumor cell-autonomous mechanisms and/or activation of the tumor microenvironment could contribute to primary and acquired erlotinib resistance, and as such, treatments based on EGFR inhibition may not be sufficient for the effective treatment of lung-cancer patients harboring mutant *EGFR*^37^. Combination of EGFR TKIs with drugs that target the IL-6R/JAK1/STAT3 pathway can be a rational synthetic lethal approach to overcome this mechanism of resistance (**Figure 1**).

Furthermore, tumor cells exposed to reversible or irreversible EGFR TKIs display early resistance dependent on MET-independent activation of BCL-2/BCL-XL survival signaling^26^. According to the study of Fan and colleagues, such cells displayed a quiescence-like state that was readily reversed after withdrawal of targeted inhibitors. BCL-2 induction and p-STAT3 (Y705) activation were found within the residual tumor cells surviving the initial antitumor response to targeted therapies^26^.

Although MAPK signaling is more commonly associated with receptor tyrosine kinases, it has been demonstrated that TGFBR1 also has tyrosine kinase activity. TGFBR1 directly phosphorylates tyrosine on ShcA, allowing for interaction with Grb2 and Sos. This leads to Ras activation, downstream ERK phosphorylation and BIM downregulation^38^. Furthermore, TGF-b can induce expression of the transcription factor Snail, which leads to repression of E-cadherin transcription. SMAD-independent activation of Snail is a result of RAS/ERK activation. Although the mechanism remains incomplete and deserves further study, in the tumor setting of aberrant TGF-b signaling, TGF-b actively represses E-cadherin and promotes EMT^38^.

More interesting is the fact that ADAM metallopeptidase domain 17 (ADAM17) or tumor necrosis factor alpha converting enzyme (TACE) targeting of TGFBR1 has been linked to ERK activation in nonprostate cells, resulting in shedding of the TGFBR1 ectodomain^38^. There is evidence that active vitamin D (1,25(OH)_2_D) therapy effectively inhibits ADAM17 and suppresses TGF/EGFR-pathway^39^. Finally, Yuan *et al*. recently demonstrated that a novel long noncoding RNA activated by TGF-b (lncRNA-ATB) amplifies the prometastatic effect of TGF-b via two independent mechanisms: firstly by inhibiting miR-200s, releasing activity of ZEB1 and ZEB2 and promoting EMT, and secondly by stabilizing *IL11*-mRNA and increasing autocrine IL-11-STAT3 signaling^40^^,^^41^.

*BIM* expression in treatment naïve cancers predicts responsiveness to EGFR TKIs, but almost 2/3 of patients have low *BIM* mRNA levels at baseline^18^. SHP2, which is downstream of both EGFR and several other tyrosine kinase receptors, is required for sustained activation of ERK and BIM downregulation^42^^,^^43^. Upon activation of MET by its ligand, hepatocyte growth factor (HGF) which is provided by stromal cells, EGFR signaling is dramatically altered^44^. Indeed, HGF enables EGFR to interact with proteins which are known to be markers of a highly metastatic phenotype like the CUB domain-containing protein-1 (CDCP1), Ephrin Type-A Receptor 2 (EphA2), but mainly AXL and Mer; these interactions cannot be affected by EGFR TKI treatment. Interestingly, STAT3 signaling can be hyperactive due to upstream pathways including not only IL-6 and JAK but also AXL, providing further opportunities for combination therapies (**Figure 1**).

## ALK NSCLC patients: second line therapy and beyond

The novel *ALK* fusion is formed by a rearrangement occurring on the short arm of chromosome 2 and involves the N-terminal portion of the EML4 protein and the intracellular signaling portion of the ALK tyrosine kinase receptor^45^. *EML4-ALK* generates a transforming tyrosine kinase with as many as nine different variants identified^46^. ALK-dependent mitogenic signaling is largely mediated via the RAS/MAP kinase pathway as well as ALK-driven PI3K activation. The JAK/STAT3 pathway also provides essential survival signals and modulates cellular metabolism regulating the mitochondrial oxidation chain. STAT3 is activated by ALK, either directly or through JAK^47^. Crizotinib, an ATP-competitive aminopyridine inhibits tyrosine phosphorylation of ALK with an IC_50_ of 20-40 nM and response in 57% of patients with ALK-rearrangement positive lung cancer^5^. However, most patients develop resistance to crizotinib, typically within one to two years. Studies of *ALK*-rearranged lung cancers with acquired resistance to crizotinib have identified *ALK* fusion gene amplification and secondary ALK TK domain mutations (L1196M and G1269A) in about one third of cases^48^.

The success of a targeted drug is critically dependent on a specific and sensitive screening assay to detect the molecular drug target. The gold standard for detection of predictive *ALK* rearrangements is currently break-apart fluorescence in situ hybridization (FISH) as it is able to detect all known *ALK*-rearrangements and was clinically validated in crizotinib clinical trials^7^. However, the ALK FISH assay is fraught with technical challenges, including FISH signal instability and scoring difficulties. Although ALK FISH is clinically validated, the assay can be technically challenging and other diagnostic modalities are being explored, including immunohistochemistry (IHC) and reverse transcriptase–polymerase chain reaction (RT-PCR). Very interestingly, high frequency of *ALK* rearrangements in thyroid cancer from atomic bomb survivors in Japan was detected by a highly sensitive RT-PCR assay from archival paraffin blocks but was not confirmed by FISH or other methods^49^. Targeted resequencing has recently been proved to be a promising method for *ALK* gene fusion detection in NSCLC, with results correlating significantly with those from FISH, RT-PCR, and IHC^50^.

As with the majority of targeted agents, the tremendous excitement and enthusiasm sparked by crizotinib is tempered by the reality that a fraction of the target tumors are refractory from time of treatment initiation and most patients will eventually relapse and develop resistance after initial response. *ALK* kinase mutations and *ALK* fusion copy number gain (CNG) have been termed ALK-dominant mechanisms of resistance since tumors harboring these mechanisms presumably preserve ALK signaling despite the presence of crizotinib and are still dependent on that pathway for survival^51^. Data from several second-generation ALK inhibitors [ceritinib (LDK378), AP26113, (alectinib)] demonstrate response rates of 55% to 60% in crizotinib-resistant ALK positive NSCLC patients, with observed or predicted disease control rates of approximately 90%^51^. Ceritinib (LDK378) is an oral, small-molecule, ATP-competitive, ALK TKI52. Ceritinib achieved 56% response rate in patients previously treated with crizotinib with various resistance mutations in *ALK*; also in patients without detectable mutations^52^. On April 29^th^ 2014, the FDA granted accelerated approval to ceritinib for treatment of patients with *ALK* positive, metastatic NSCLC with disease progression on or intolerant to crizotinib. The approval of ceritinib was based on the results of a multicenter, single-arm, open-label clinical trial enrolling 163 patients with metastatic *ALK*-positive NSCLC who had progressed on or were intolerant to crizotinib. This study is part of the ongoing clinical trial program in this patient population. Zykadia achieved an ORR of 54.6% and a median duration of response of 7.4 months based on investigator assessment^53^. However, while ceritinib is able to effectively suppress many crizotinib-resistant mutations, the G1202R and F1174V/C mutants are resistant to ceritinib^48^.

One of the questions surrounding second-generation ALK inhibitors is exactly how they will fit into the treatment landscape of *ALK* positive NSCLC and whether they stand a chance of outshining crizotinib at the frontline. Furthermore, encouraging early clinical results in NSCLC have demonstrated that ganetespib, a novel triazolone inhibitor of HSP90, may offer a potential strategy to target *ALK* inhibition by inducing substantial antitumor responses and overcoming acquired resistance in patients with *ALK* positive lung cancer. When combined with ganetespib, crizotinib displayed superior antitumor efficacy compared with monotherapy in H3122 NSCLC xenografts^54^. Recently, Richards *et al*. found that sensitivity of *EML4-ALK* variants to HSP90 inhibitors differs depending on whether the breakpoint of the fusion protein interrupts the globular TAPE (tandem atypical propeller in EMLs) domain. They determined the molecular structure of a conserved, tubulin-binding region of *EML1* that reveals an unexpected protein fold^55^. This region is disrupted in almost 70% of *EML4-ALK* fusions found in patients, causing them to be sensitive to drugs that target HSP90, a cellular factor that stabilizes misfolded protein. These findings suggest that the truncation of a globular domain at the translocation breakpoint may prove generally predictive of HSP90 inhibitor sensitivity in cancers driven by fusion oncogenes^55^.

Other “*ALK*-independent” mechanisms, such as the activation of compensatory signaling pathways, may also confer resistance to targeted ALK agents. Bypass signaling, by v-Kit Hardy-Zuckerman 4 feline sarcoma viral oncogene homolog (*KIT*) or *EGFR*, has also been described as a mechanism of resistance to crizotinib in *ALK* positive NSCLC^51^. EGF-induced activation of EGFR, HER2, and HER3, as well as a reduced level of ALK activation, was associated with sustained downstream signaling in the presence of ALK inhibitors, indicative of a shift in survival dependency from the ALK signaling pathway to HER family pathways in ALK-TKI–resistant cells^56^. The combination of an ALK inhibitor and an EGFR inhibitor induced apoptosis, further supporting the notion that the EGFR signaling pathway contributes to survival in cells resistant to ALK inhibitors^56^. *ALK* rearrangements and *EGFR* mutations could coexist in a small subgroup of NSCLC. Advanced pulmonary adenocarcinomas with such co-alterations could have diverse responses to EGFR-TKIs and crizotinib^57^. Relative phospho-ALK and phospho-EGFR levels could predict the efficacy of EGFR-TKI and crizotinib. Yang *et al*. recently described co-altered *EGFR* and *ALK* in a large cohort of NSCLC and found that 3.9% of *EGFR* mutant and 18.6% of *ALK* rearranged tumors have co-alterations^57^. Tumors harboring co-altered *EGFR* and *ALK* could have diverse responses to first-line EGFR-TKIs, which were associated with phospho-EGFR levels^57^. Phospho-ALK levels correlated with efficacy of subsequent crizotinib treatment. In clinical practice, we should pay attention to the specific biological behavior and corresponding management of NSCLC with dual altered EGFR and ALK genes^57^.

**Figure 2:**
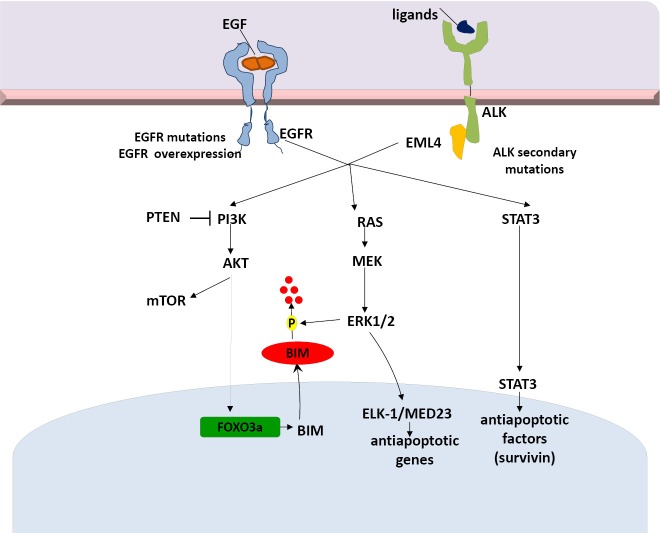
EML4-ALK signaling pathways and potential mechanisms of resistance to targeted therapies. EML4-ALK, echinoderm microtubule-associated protein-like 4- anaplastic lymphoma kinase.

ALK and its ligand pleiotrophin are required for self-renewal and tumorigenicity of glioblastoma stem cells (GSCs)^58^. The pleiotrophin-ALK axis is activated by SOX2 and may be a promising target for therapy in ALK signaling-dependent tumors^58^. Finally, Takezawa *et al*. demonstrated that expression of BIM and survivin are independently regulated by ERK and STAT3 signaling pathways, respectively, and are implicated in ALK-TKI-induced apoptosis in NSCLC cells positive for EML4-ALK^59^. A selective inhibitor of ALK kinase activity (more potent than crizotinib), NVP-TAE68, inhibits STAT3 phosphorylation and downregulates survivin in H2228 cells, but fails to inhibit ERK phosphorylation and upregulate BIM. In a recent study, inhibition of both STAT3 and ERK pathways with the combination of TAE684 and a MEK inhibitor, AZD6244, was associated with a marked increase in the number of apoptotic cells^60^ (**Figure 2**).

## Conclusion

TKIs are effective anti-cancer therapies but resistance to these agents eventually develops. Several models of resistance to TKIs have been studied, including resistance to EGFR inhibitors or ALK inhibitors. A decade after the discovery of *EGFR* mutations, treatment is still incomplete and growing evidence indicates that targeting STAT3 or upstream or downstream components could cause induce synthetic lethal effects and achieve better outcomes. Repurposing drugs in combination with gefitinib, erlotinib, afatinib and dacomitinib is being investigated in cell cultures, subcutaneous & orthotopic xenograft models and clinical tumor samples. Clearly, the use of oncogene-addicted, highly drug-sensitive cell line models to identify escape mechanisms should be a strategy for identifying and overcoming resistance. However, it is of paramount importance that whenever possible, a biopsy is taken at time of cancer recurrence in patients who become resistant to targeted therapies so that these mechanisms can be confirmed or refuted. By identifying how a patient’s cancer becomes refractory to targeted therapies, we will be well positioned to design rational treatment strategies to re-induce remissions. Only with expanding knowledge of these escape mechanisms, availability of drugs targeting escape pathways, and examples of such molecular data informing treatment decisions resulting in good clinical outcome, can re-biopsy and molecular profiling of recurrent drug-resistant tumors be widely incorporated into clinical practice.

## References

[1] Rosell R Bivona TG Karachaliou N Genetics and biomarkers in personalisation of lung cancer treatment. Lancet 2013;382:720-731 23972815 10.1016/S0140-6736(13)61715-8

[2] Rosell R Carcereny E Gervais R Vergnenegre A Massuti B Felip E Erlotinib versus standard chemotherapy as first-line treatment for European patients with advanced EGFR mutation-positive non-small-cell lung cancer (EURTAC): a multicentre, open-label, randomised phase 3 trial. Lancet Oncol 2012;13:239-246 22285168 10.1016/S1470-2045(11)70393-X

[3] Soda M Choi YL Enomoto M Takada S Yamashita Y Ishikawa S Identification of the transforming EML4-ALK fusion gene in non-small-cell lung cancer. Nature 2007;448:561-566 17625570 10.1038/nature05945

[4] Sasaki T Rodig SJ Chirieac LR Jänne PA The biology and treatment of EML4-ALK non-small cell lung cancer. Eur J Cancer 2010;46:1773-1780 20418096 10.1016/j.ejca.2010.04.002 PMC2888755

[5] Kwak EL Bang YJ Camidge DR Shaw AT Solomon B Maki RG Anaplastic lymphoma kinase inhibition in non-small-cell lung cancer. N Engl J Med 2010;363:1693-1703 20979469 10.1056/NEJMoa1006448 PMC3014291

[6] Crino L, Kim D, Riely GJ, Janne PA, Blackhall FH, Camidge DR, et al. Initial phase II results with crizotinib in advanced ALK-positive non-small cell lung cancer (NSCLC): profile 1005. ASCO Meet Abstr 2011;29:7514.

[7] Camidge DR Bang YJ Kwak EL Iafrate AJ Varella-Garcia M Fox SB Activity and safety of crizotinib in patients with ALK-positive non-small-cell lung cancer: updated results from a phase 1 study. Lancet Oncol 2012;13:1011-1019 22954507 10.1016/S1470-2045(12)70344-3 PMC3936578

[8] Rosell R Moran T Queralt C Porta R Cardenal F Camps C Screening for epidermal growth factor receptor mutations in lung cancer. N Engl J Med 2009;361:958-967 19692684 10.1056/NEJMoa0904554

[9] Mok TS Wu YL Thongprasert S Yang CH Chu DT Saijo N Gefitinib or carboplatin-paclitaxel in pulmonary adenocarcinoma. N Engl J Med 2009;361:947-957 19692680 10.1056/NEJMoa0810699

[10] Garuti L Roberti M Bottegoni G. Irreversible protein kinase inhibitors. Curr Med Chem 2011;18:2981-2994 21651479 10.2174/092986711796391705

[11] Ou SH Second-generation irreversible epidermal growth factor receptor (EGFR) tyrosine kinase inhibitors (TKIs): a better mousetrap? A review of the clinical evidence. Crit Rev Oncol Hematol 2012;83:407-421 22257651 10.1016/j.critrevonc.2011.11.010

[12] Ninomiya T Takigawa N Ichihara E Ochi N Murakami T Honda Y Afatinib prolongs survival compared with gefitinib in an epidermal growth factor receptor-driven lung cancer model. Mol Cancer Ther 2013;12:589-597 23443806 10.1158/1535-7163.MCT-12-0885

[13] Sequist LV Yang JC Yamamoto N O’Byrne K Hirsh V Mok T Phase III study of afatinib or cisplatin plus pemetrexed in patients with metastatic lung adenocarcinoma with EGFR mutations. J Clin Oncol 2013;31:3327-3334 23816960 10.1200/JCO.2012.44.2806

[14] Yang JC, Sequist LV, Schuler MH, Mok T, Yamamoto N, O’Byrne KJ, et al. Overall survival (OS) in patients (pts) with advanced non-small cell lung cancer (NSCLC) harboring common (Del19/L858R) epidermal growth factor receptor mutations (EGFR mut): Pooled analysis of two large open-label phase III studies (LUX-Lung 3 [LL3] and LUX-Lung 6 [LL6]) comparing afatinib with chemotherapy (CT). J Clin Oncol 2014;32:abstr 8004.

[15] Lee CK Brown C Gralla RJ Hirsh V Thongprasert S Tsai CM Impact of EGFR inhibitor in non-small cell lung cancer on progression-free and overall survival: a meta-analysis. J Natl Cancer Inst 2013;105:595-605 23594426 10.1093/jnci/djt072

[16] Sequist LV Waltman BA Dias-Santagata D Digumarthy S Turke AB Fidias P Genotypic and histological evolution of lung cancers acquiring resistance to EGFR inhibitors. Sci Transl Med 2011;3:75ra26 21430269 10.1126/scitranslmed.3002003 PMC3132801

[17] Spigel DR, Edelman MJ, O’Byrne K, Paz-Ares L, Shames DS, Yu W, et al. Onartuzumab plus erlotinib versus erlotinib in previously treated stage IIIb or IV NSCLC: Results from the pivotal phase III randomized, multicenter, placebo-controlled METLung (OAM4971g) global trial. J Clin Oncol 2014;32:abstr 8000.

[18] Costa C Molina MA Drozdowskyj A Giménez-Capitán A Bertran-Alamillo J Karachaliou N The impact of EGFR T790M mutations and BIM mRNA expression on outcome in patients with EGFR-mutant NSCLC treated with erlotinib or chemotherapy in the randomized phase III EURTAC trial. Clin Cancer Res 2014;20:2001-2010 24493829 10.1158/1078-0432.CCR-13-2233

[19] Faehling M Eckert R Kamp T Kuom S Griese U Sträter J EGFR-tyrosine kinase inhibitor treatment beyond progression in long-term Caucasian responders to erlotinib in advanced non-small cell lung cancer: a case-control study of overall survival. Lung Cancer 2013;80:306-312 23489557 10.1016/j.lungcan.2013.02.010

[20] Kim SM Kwon OJ Hong YK Kim JH Solca F Ha SJ Activation of IL-6R/JAK1/STAT3 signaling induces de novo resistance to irreversible EGFR inhibitors in non-small cell lung cancer with T790M resistance mutation. Mol Cancer Ther 2012;11:2254-2264 22891040 10.1158/1535-7163.MCT-12-0311

[21] Walter AO Sjin RT Haringsma HJ Ohashi K Sun J Lee K Discovery of a mutant-selective covalent inhibitor of EGFR that overcomes T790M-mediated resistance in NSCLC. Cancer Discov 2013;3:1404-1415 24065731 10.1158/2159-8290.CD-13-0314 PMC4048995

[22] Berardi R Santoni M Morgese F Ballatore Z Savini A Onofri A Novel small molecule EGFR inhibitors as candidate drugs in non-small cell lung cancer. Onco Targets Ther 2013;6:563-576 23723712 10.2147/OTT.S28155 PMC3665567

[23] Sequist LV, Soria JC, Gadgeel SM, Wakelee HA, Camidge DR, Varga A, et al. First-in-human evaluation of CO-1686, an irreversible, highly selective tyrosine kinase inhibitor of mutations of EGFR (activating and T790M). J Clin Oncol 2014;32:abstr 8010.

[24] Targeting Resistance in Lung Cancer Cancer Discov 2013;3:OF9 10.1158/2159-8290.CD-ND2013-025 24327718

[25] Janne PA, Ramalingam SS, Yang JC, Ahn MJ, Kim DW, Kim SW, et al. Clinical activity of the mutant-selective EGFR inhibitor AZD9291 in patients (pts) with EGFR inhibitor–resistant non-small cell lung cancer (NSCLC). J Clin Oncol 2014;32:abstr 8009.

[26] Fan W Tang Z Yin L Morrison B Hafez-Khayyata S Fu P MET-independent lung cancer cells evading EGFR kinase inhibitors are therapeutically susceptible to BH3 mimetic agents. Cancer Res 2011;71:4494-4505 21555370 10.1158/0008-5472.CAN-10-2668 PMC3132557

[27] Ng KP Hillmer AM Chuah CT Juan WC Ko TK Teo AS A common BIM deletion polymorphism mediates intrinsic resistance and inferior responses to tyrosine kinase inhibitors in cancer. Nat Med 2012;18:521-528 22426421 10.1038/nm.2713

[28] Nakagawa T Takeuchi S Yamada T Ebi H Sano T Nanjo S EGFR-TKI resistance due to BIM polymorphism can be circumvented in combination with HDAC inhibition. Cancer Res 2013;73:2428-2434 23382048 10.1158/0008-5472.CAN-12-3479

[29] Dasari VR Velpula KK Alapati K Gujrati M Tsung AJ Cord blood stem cells inhibit epidermal growth factor receptor translocation to mitochondria in glioblastoma. PloS one 2012;7:e31884 22348136 10.1371/journal.pone.0031884 PMC3279427

[30] Yu B Liu W Yu WM Loh ML Alter S Guvench O Targeting protein tyrosine phosphatase SHP2 for the treatment of PTPN11-associated malignancies. Mol Cancer Ther 2013;12:1738-1748 23825065 10.1158/1535-7163.MCT-13-0049-T PMC3769526

[31] You M Yu DH Feng GS Shp-2 tyrosine phosphatase functions as a negative regulator of the interferon-stimulated Jak/STAT pathway. Mol Cell Biol 1999;19:2416-2424 10022928 10.1128/mcb.19.3.2416 PMC84034

[32] Sordella R Bell DW Haber DA Settleman J Gefitinib-sensitizing EGFR mutations in lung cancer activate anti-apoptotic pathways. Science 2004;305:1163-1167 15284455 10.1126/science.1101637

[33] Lazzara MJ Lane K Chan R Jasper PJ Yaffe MB Sorger PK Impaired SHP2-mediated extracellular signal-regulated kinase activation contributes to gefitinib sensitivity of lung cancer cells with epidermal growth factor receptor-activating mutations. Cancer Res 2010;70:3843-3850 20406974 10.1158/0008-5472.CAN-09-3421 PMC2862125

[34] Li L Han R Xiao H Lin C Wang Y Liu H Metformin sensitizes EGFR-TKI-resistant human lung cancer cells in vitro and in vivo through inhibition of IL-6 signaling and EMT reversal. Clin Cancer Res 2014;20:2714-2726 24644001 10.1158/1078-0432.CCR-13-2613

[35] Alvarez JV Greulich H Sellers WR Meyerson M Frank DA Signal transducer and activator of transcription 3 is required for the oncogenic effects of non-small-cell lung cancer-associated mutations of the epidermal growth factor receptor. Cancer Res 2006;66:3162-3168 16540667 10.1158/0008-5472.CAN-05-3757

[36] Gao SP Mark KG Leslie K Pao W Motoi N Gerald WL Mutations in the EGFR kinase domain mediate STAT3 activation via IL-6 production in human lung adenocarcinomas. J Clin Invest 2007;117:3846-3856 18060032 10.1172/JCI31871 PMC2096430

[37] Yao Z Fenoglio S Gao DC Camiolo M Stiles B Lindsted T TGF-beta IL-6 axis mediates selective and adaptive mechanisms of resistance to molecular targeted therapy in lung cancer. Proc Natl Acad Sci U S A 2010;107:15535-15540 20713723 10.1073/pnas.1009472107 PMC2932568

[38] Principe DR, Doll JA, Bauer J, Jung B, Munshi HG, Bartholin L, et al. TGF-beta: duality of function between tumor prevention and carcinogenesis. J Nat Cancer Institute 2014;106:djt369. 10.1093/jnci/djt369 PMC3952197 24511106

[39] Dusso A Arcidiacono MV Yang J Tokumoto M Vitamin D inhibition of TACE and prevention of renal osteodystrophy and cardiovascular mortality. J Steroid Biochem Mol Biol 2010;121:193-198 20359533 10.1016/j.jsbmb.2010.03.064 PMC2906659

[40] Li W Kang Y. A new Lnc in metastasis: long noncoding RNA mediates the prometastatic functions of TGF-β. Cancer Cell 2014;25:557-559 24823634 10.1016/j.ccr.2014.04.014 PMC4091806

[41] Yuan JH Yang F Wang F Ma JZ Guo YJ Tao QF A long noncoding RNA activated by TGF-β promotes the invasion-metastasis cascade in hepatocellular carcinoma. Cancer Cell 2014;25:666-681 24768205 10.1016/j.ccr.2014.03.010

[42] Miura K Wakayama Y Tanino M Orba Y Sawa H Hatakeyama M Involvement of EphA2-mediated tyrosine phosphorylation of Shp2 in Shp2-regulated activation of extracellular signal-regulated kinase. Oncogene 2013;32:5292-5301 23318428 10.1038/onc.2012.571

[43] Maroun CR Naujokas MA Holgado-Madruga M Wong AJ Park M The tyrosine phosphatase SHP-2 is required for sustained activation of extracellular signal-regulated kinase and epithelial morphogenesis downstream from the met receptor tyrosine kinase. Mol Cell Biol 2000;20:8513-8525 11046147 10.1128/mcb.20.22.8513-8525.2000 PMC102157

[44] Gusenbauer S Vlaicu P Ullrich A. HGF induces novel EGFR functions involved in resistance formation to tyrosine kinase inhibitors. Oncogene 2013;32:3846-3856 23045285 10.1038/onc.2012.396

[45] Shaw AT Hsu PP Awad MM Engelman JA Tyrosine kinase gene rearrangements in epithelial malignancies. Nat Rev Cancer 2013;13:772-787 24132104 10.1038/nrc3612 PMC3902129

[46] Puig de la Bellacasa R Karachaliou N Estrada-Tejedor R Teixidó J Costa C Borrell JI ALK and ROS1 as a joint target for the treatment of lung cancer: a review. Transl Lung Cancer Res 2013;2:72-86 10.3978/j.issn.2218-6751.2013.03.1 PMC4369855 25806218

[47] Barreca A Lasorsa E Riera L Machiorlatti R Piva R Ponzoni M Anaplastic lymphoma kinase in human cancer. J Mol Endocrinol 2011;47:R11-R23 21502284 10.1530/JME-11-0004

[48] Friboulet L Li N Katayama R Lee CC Gainor JF Crystal AS The ALK inhibitor ceritinib overcomes crizotinib resistance in non-small cell lung cancer. Cancer Discov 2014;4:662-673 24675041 10.1158/2159-8290.CD-13-0846 PMC4068971

[49] Kelly LM Barila G Liu P Evdokimova VN Trivedi S Panebianco F Identification of the transforming STRN-ALK fusion as a potential therapeutic target in the aggressive forms of thyroid cancer. Proc Natl Acad Sci U S A 2014;111:4233-4238 24613930 10.1073/pnas.1321937111 PMC3964116

[50] Tuononen K, Sarhadi VK, Wirtanen A, Rönty M, Salmenkivi K, Knuuttila A, et al. Targeted resequencing reveals ALK fusions in non-small cell lung carcinomas detected by FISH, immunohistochemistry, and real-time RT-PCR: a comparison of four methods. Biomed Res Int 2013;2013:757490. 10.1155/2013/757490 PMC3581296 23484153

[51] Doebele RC A nice problem to have: when ALK inhibitor therapy works better than expected. J Thorac Oncol 2014;9:433-435 24736061 10.1097/JTO.0000000000000124

[52] Shaw AT Kim DW Mehra R Tan DS Felip E Chow LQ Ceritinib in ALK-rearranged non-small-cell lung cancer. N Engl J Med 2014;370:1189-1197 24670165 10.1056/NEJMoa1311107 PMC4079055

[53] Information ZTcP. East Hanover, New Jersey, USA: Novartis Pharmaceuticals Corporation. April 2014.

[54] Sang J Acquaviva J Friedland JC Smith DL Sequeira M Zhang C Targeted inhibition of the molecular chaperone Hsp90 overcomes ALK inhibitor resistance in non-small cell lung cancer. Cancer Discov 2013;3:430-443 23533265 10.1158/2159-8290.CD-12-0440 PMC4086149

[55] Richards MW Law EW Rennalls LP Busacca S O’Regan L Fry AM Crystal structure of EML1 reveals the basis for Hsp90 dependence of oncogenic EML4-ALK by disruption of an atypical β-propeller domain. Proc Natl Acad Sci U S A 2014;111:5195-5200 24706829 10.1073/pnas.1322892111 PMC3986153

[56] Tanizaki J Okamoto I Okabe T Sakai K Tanaka K Hayashi H Activation of HER family signaling as a mechanism of acquired resistance to ALK inhibitors in EML4-ALK-positive non-small cell lung cancer. Clin Cancer Res 2012;18:6219-6226 22843788 10.1158/1078-0432.CCR-12-0392

[57] Yang JJ Zhang XC Su J Xu CR Zhou Q Tian HX Lung cancers with concomitant EGFR mutations and ALK rearrangements: diverse responses to EGFR-TKI and crizotinib in relation to diverse receptors phosphorylation. Clin Cancer Res 2014;20:1383-1392 24443522 10.1158/1078-0432.CCR-13-0699

[58] Koyama-Nasu R Haruta R Nasu-Nishimura Y Taniue K Katou Y Shirahige K The pleiotrophin-ALK axis is required for tumorigenicity of glioblastoma stem cells. Oncogene 2014;33:2236-2244 23686309 10.1038/onc.2013.168

[59] Takezawa K Okamoto I Nishio K Jänne PA Nakagawa K Role of ERK-BIM and STAT3-survivin signaling pathways in ALK inhibitor-induced apoptosis in EML4-ALK-positive lung cancer. Clin Cancer Res 2011;17:2140-2148 21415216 10.1158/1078-0432.CCR-10-2798

[60] Tanizaki J Okamoto I Takezawa K Sakai K Azuma K Kuwata K Combined effect of ALK and MEK inhibitors in EML4-ALK-positive non-small-cell lung cancer cells. Br J Cancer 2012;106:763-767 22240786 10.1038/bjc.2011.586 PMC3322944

